# Effect of static magnetic field therapy on diabetic neuropathy and quality of life: a double-blind, randomized trial

**DOI:** 10.1186/s13098-023-01123-9

**Published:** 2023-07-04

**Authors:** Armin Nazeri, Ali Mohammadpour, Mohammad-Hadi Saeed Modaghegh, Mojtaba Kianmehr

**Affiliations:** 1 Student Research Committee, Gonabad University of Medical Sciences, Gonabad, Iran; 2 Department of Nursing, School of Nursing, Social Determinants of Health Research Center, Gonabad University of Medical Sciences, Gonabad, Iran; 3 Vascular and Endovascular Surgery Research Center, Mashhad University of Medical Sciences, Mashhad, Iran; 4 Department of Medical Physics and Radiology, School of Paramedicine, Social Development and Health Promotion Research Center, Gonabad University of Medical Sciences, Gonabad, Iran

**Keywords:** Magnetic field therapy, Diabetes mellitus, type 2, Diabetic neuropathies, Pain, Quality of life

## Abstract

**Background:**

Diabetic peripheral neuropathy (DPN) is a common complication of diabetes mellitus (DM) that can cause annoying symptoms. To address this condition, several treatment approaches have been proposed, including static magnetic field (SMF) therapy, which has shown promise in treating neurological conditions. Therefore, this study aimed to investigate the effects of SMF therapy on symptomatic DPN and the quality of life (QoL) in patients with type 2 diabetes.

**Methods:**

A double-blind, randomized, placebo-controlled trial was conducted from April to October 2021. Sixty-four DPN patients (20 males, 44 females) were recruited for the study via invitation. The participants were divided into two groups: the magnet group, which used magnetic ankle bracelets (155 mT) for 12 weeks, and the sham group, which used non-magnetic ankle bracelets for the same duration. Neuropathy Symptom Score (NSS), Neuropathic Disability Score (NDS), and Visual Analogue Scale (VAS) were used to assess neuropathy symptoms and pain. In addition, the Neuropathy Specific Quality of Life Questionnaire (Neuro-QoL) tool was used to measure the patients’ quality of life.

**Results:**

Before treatment, there were no significant differences between the magnet and sham groups in terms of the NSS scores (P = 0.50), NDS scores (P = 0.74), VAS scores (P = 0.17), and Neuro-QoL scores (P = 0.82). However, after 12 weeks of treatment, the SMF exposure group showed a significant reduction in NSS scores (P < 0.001), NDS scores (P < 0.001), VAS scores (P < 0.001), and Neuro-QoL scores (P < 0.001) compared to the baseline. The changes in the sham group, on the other hand, were not significant.

**Conclusion:**

According to obtained data, SMF therapy is recommended as an easy-to-use and drug-free method for reducing DPN symptoms and improving QoL in diabetic type-2 patients.

*Trial registration* Registered at Iranian Registry of Clinical Trials: IRCT20210315050706N1, 2021/03/16.

## Introduction

Diabetic peripheral neuropathy (DPN) is the most common and serious long-term complication of diabetes, and it is associated with increased mortality and morbidity among diabetic patients [1]. According to previous studies, DPN affects nearly 50–90% of diabetic patients [2–4]. DPN can lead to several complications, such as foot ulcers, Charcot arthropathy, lower extremity amputation, and increased healthcare costs among patients with diabetes [5, 6]. Selective involvement of unmyelinated C fibers and small myelinated A-delta fibers produces pain of the burning synesthetic type and it is often accompanied by hyperalgesia and allodynia in the feet [7]. Similarly, various DPN symptoms, such as burning, tingling, numbness, pins-needles sensations, tightness, itchiness, sensory ataxia, and neuropathic pain have been reported [8]. Based on clinical guidelines recommendation, although pharmacotherapy is a common and significantly beneficial method for symptomatic DPN relief such as tricyclic antidepressants (TCAs), anticonvulsants, and narcotic analgesics, frequent use of this drug results in significant health side effects [9, 10]. According to current international guidelines, physicians should offer a tricyclic antidepressant, duloxetine, or gabapentinoid as first-line mono-pharmacotherapy for treatment. The choice of first-line treatment depends on the comorbidities and contraindications of the patients [11]. TCAs are contraindicated in patients with cardiovascular disease including ischaemic heart disease and arrhythmias. Gabapentinoids may be avoided in patients with heart failure and/or peripheral edema. Duloxetine is cautioned with co-existing GI symptoms, eg, bloating, nausea, and dizziness as these symptoms may be exacerbated [11]. These main drugs, which are used in the treatment of diabetic peripheral neuropathy, have limitations on their use due to side effects, drug interactions, and contraindications in some diseases. Common adverse events of pregabalin include dizziness, weight gain, peripheral edema, headache, and drowsiness. Common adverse events with the use of TCAs include drowsiness, dizziness, headaches, drowsiness, dry mouth, constipation, nausea, arrhythmias, and orthostatic hypotension. TCAs are contraindicated in cardiovascular diseases such as arrhythmias, severe hepatic impairment, patients with urinary retention, orthostatic hypotension, and constipation. Common adverse events of duloxetine include headaches, nausea, dry mouth, and drowsiness. Nausea, drowsiness, headache, vertigo, dizziness, and constipation are opioids’ common adverse events [11–13]. Given the health side effects and limitations on their use in DPN, as well as their limited availability in some countries, alternative and complementary medicine can be a viable solution. Nowadays, patients prefer safe and effective new non-pharmacologic therapies over pharmacologic treatments. Therefore, current treatment recommendations suggest combining existing therapies or using them in isolation [8, 9, 14]. Furthermore, there is a growing tendency towards alternative and complementary treatments in medical science. However, reliable, safe, and effective mainstream treatments for neuropathic pain remain a question. This challenge has led patients to explore different alternative approaches, such as homeopathy, acupuncture, and magnetic therapies. In this context, it does not come as a surprise that the use of permanent magnets for the relief of pain has become extremely popular in the diabetic patient community. Magnetic therapy is one of the most intriguing combination treatment methods for DPN treatment. However, the static magnetic field (SMF) is a non-invasive physical tool and is considered the most important field in magnetic therapy. Animal-based studies have reported that SMF can help improve pain and wound healing and has a protective role in diabetic mice [15–17]. Furthermore, several studies have demonstrated that Randomized Controlled Trials (RCTs) effectively treat various disorders in patients, such as carpal tunnel syndrome and arthritis [18–20]. In this regard, three biophysical mechanisms describe potential interactions between living tissue and SMF: (a) electronic interactions, (b) magneto-mechanical effects, and (c) forces on moving charged particles [21]. Some proposed theories include increased blood flow changes in the dynamics of calcium ions, and nociceptive C fibers [21–25], however, the underlying mechanisms are presently unclear. SMF therapy is considered a safe, non-invasive, drug-free, and durable intervention with few reported side effects. Moreover, it is relatively simple to operate, making it an attractive treatment option for patients with DPN [15, 17, 22, 23]. Previous studies have reported that specific sources of magnetic fields, such as the SMF and pulsed electromagnetic fields (PEMFs), may have beneficial effects in treating or preventing diabetes. A study conducted by Weintraub and colleagues found that exposure to a 45 mT SMF reduced neuropathic symptoms in diabetic patients [26]. Chronic neuropathic pain and other symptoms associated with DPN can significantly decrease the quality of life (QoL) of affected individuals [27–29]. Therefore, improving QoL is one of the main objectives of clinical care for DPN patients. This can be achieved through various interventions, including non-pharmacologic therapies such as SMF therapy, as well as pharmacologic treatments and lifestyle modifications. Previous research has demonstrated that applying local magnetic energy to the feet can have a positive effect on chronic neuropathic pain treatment. However, without randomized, placebo-controlled trials, the medical community cannot accept magnets as a valid option for pain relief. Therefore, the present study was designed as a nationwide double-blind placebo-controlled trial to investigate the efficacy of static magnetic fields in treating DPN and its impact on patients' quality of life.

## Materials and methods

### Study design

The present research study was designed as a randomized, double-blind, placebo-controlled trial conducted from April 1, 2021, to October 30, 2021, at Alavi Hospital in Mashhad, Razavi Khorasan, Iran. The 4:4 blocks method was used for randomization. The trial was approved by the Ethics Committee of Gonabad University of Medical Sciences (approval number: IR.GMU.REC.1399.132) and registered at the Iranian Registry of Clinical Trials (IRCT20210315050706N1).

### Participant

The study included 64 participants who were recruited from the Diabetes Clinic at Alavi Hospital in Mashhad, Iran, between April 21, 2021, and July 27, 2021. Participants were invited to participate in the study by their physicians or through phone calls, based on pre-determined inclusion and exclusion criteria (see Table 1). Participants were then randomized into two groups, and a magnetic device was randomly assigned to each participant in a double-blind manner.

**Table 1:** Inclusion/exclusion criteria

Inclusion criteria	Exclusion criteria
Diabetes type 2; diagnosed by a diabetologist according to ADA standards	Pregnancy, planning a pregnancy, lactation
DPN at least 6 scores on score NDS without NSS score or 3–5 scores on NDS with at least 5 scores on NSS	Had any of the following: Vascular insufficiency, Renal failure, Metallic implantation, Skin diseases, Foot ulcers, Prosthesis, Prior magnetic therapy, Cardiac pacemaker, Mechanical insulin pump or any electronic device
Drug-refractory neuropathic pain intensity at least 1 score on VAS	Had DPN from other causes than diabetes (according to the medical history and diagnosis of specialist doctors)
Aged 18–70 years (either sex)	Opiate or drug abuse
Able to complete questionnaires and willing to sign written informed consent	

### Magnetic device

**Fig. 1:**
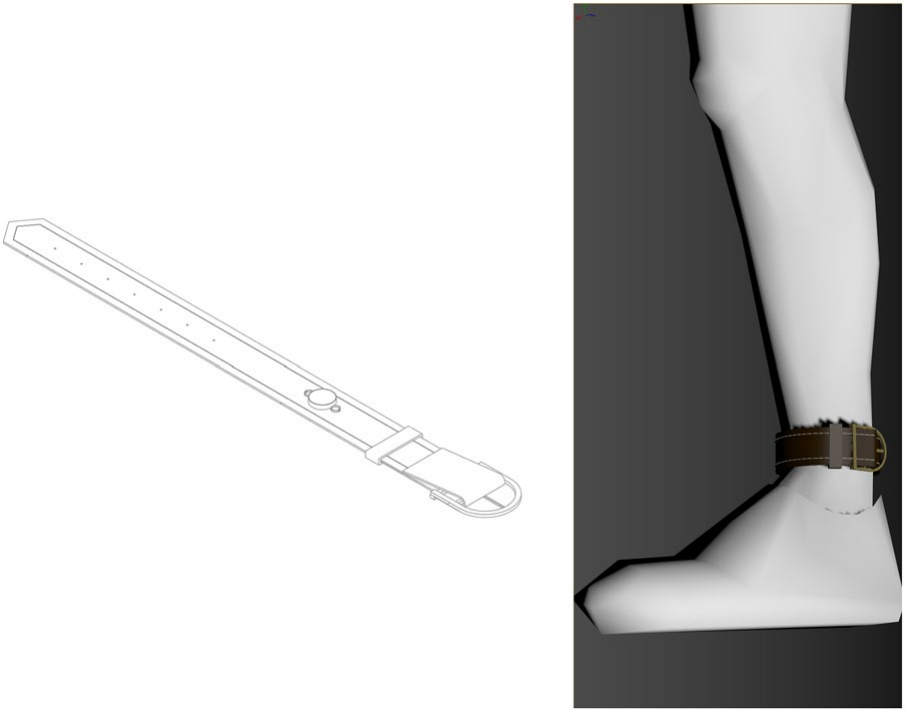
Magnetic ankle bracelet, consisting of a leather wrist strap and a disc-shaped neodymium-iron-boron insert

A disc neodymium-iron-boron (NdFeB) was used to produce the static magnetic field. The magnetic flux density was measured using a Tesla meter (Model GmbH; LD Didactic, Germany) with a transversal Hall Probe (Axial B-Sonde S, LD Didactic) with a sensitivity of 0.01 mT. The magnetic field intensity was measured at 155 mT and 66.8 mT in the center and lowest area, respectively. The magnets used in the study measured 15 mm (length), 15 mm (width), and 3 mm (height) and were placed in a leather ankle bracelet with the South Pole magnet in contact with the skin (see Fig. 1).

### Sham device

The sham device used in the study was designed to look identical to the magnetic device but was non-magnetic, as confirmed by a Hall probe measurement. Both the sham and magnetic devices were applied to patients in a similar manner. Participants were instructed to wear the devices constantly, 24 h per day, except during bathing, throughout the research period. To control for other factors that may have affected the results, plasma glucose concentrations were measured 8 times a month, including measurements taken in a fasting state, after breakfast, after lunch, and after dinner (twice2hpp).

### Outcome measures

The primary outcome measures include: (a) Assessment of neuropathy symptoms using the Neuropathy Symptom Score (NSS). The NSS assesses several symptoms, including burning, numbness, paresthesia, weakness (including fatigue and exhaustion), cramps, pain, localization symptoms on feet or elsewhere, exacerbation at night or day, and waking up from sleep due to the symptoms. It also assesses symptom improvement when walking, standing, or sitting. The score ranges from 0 to 16, with scores of 3–4 indicating mild neuropathic symptoms, 5–6 indicating moderate symptoms, and scores ≥ 7 indicating severe neuropathic symptoms [30, 31], (b) Assessment of pain severity using the Visual Analogue Scale (VAS). The VAS is a valid and reliable instrument for assessing perceived pain. It consists of a 100 mm horizontal line labeled ‘no pain’ on the left (i.e., 0 mm) and ‘worst possible pain’ on the right (i.e., 100 mm) [32]. The primary outcome measures of this study were the differences in neuropathy symptom scores and pain scores at week 12 compared to baseline scores. We also analyzed month-to-month changes in these scores. The secondary outcomes of this study include: (a) Assessment of neuropathic disability using the Neuropathic Disability Score (NDS). The NDS assesses several factors, including temperature perception, vibratory sensibility measurement dorsal on the big toe joint using a 128 Hz tuning fork, pinprick sensation measurement on the dorsum of the foot, and Achilles' reflex from a relaxed sitting position. The score ranges from 0 to 10, with scores of 3–5 indicating mild neuropathic disability, 6–8 indicating moderate disability, and scores of 9–10 indicating severe disability. Each foot is scored separately, and abnormal scores are indicated by a score of 1 or 2 [30, 31], (b) Assessment of quality of life using the Neuro-Qol tool. The Neuro-Qol tool is a validated instrument for assessing the quality of life in patients with neurological disorders. It includes several domains, such as physical functioning, emotional functioning, and social functioning, and provides a comprehensive assessment of the patient's health-related quality of life [33]. The secondary outcome score of this study is the differences in neuropathic disability scores and quality of life scores at week 12 compared to baseline scores.

### Sample size

Based on the previous study, to attain 90% power and 95% confidence intervals for each group, we determined that a sample size of 25 participants would be required [34]. Therefore, the sample size was increased to 32 participants in each group for covering potential dropouts.

### Randomization and blinding

Patients were selected for the study using purposeful sampling, based on the inclusion criteria. Referrals to the clinic were screened to identify patients who met the inclusion criteria. To ensure that the study groups were balanced and comparable at baseline, the intervention group (A) and the sham group (B) were randomized using the quadruple block method. In this study, six possible block arrangements (AABB, BBAA, ABAB, BABA, ABBA, BAAB) were listed, and one number from 1 to 6 was assigned to each block. One number (between 1 and 6) was then randomly selected, and individuals were assigned to groups (A) and (B) based on the respective block. This process was repeated until the sample size was completed. To minimize bias in the study, both patients and researchers were blinded to the intervention. A research assistant assigned patients to the groups randomly and selected the appropriate device. There was no distinguishable difference between the sham and SMF devices in terms of their appearance, weight, or texture, which helped to ensure that the study was double-blinded. To further reduce bias in the study outcomes, patients were informed that the lack of an acute change in neuropathy symptoms and pain did not necessarily mean that they had received a sham device. This helped to minimize the potential for placebo effects.

### Statistical analysis

Outcomes were analyzed for normal distributions by using the Kolmogorov–Smirnov test. To compare the demographic data of the two groups, chi-square analysis or Fisher's exact test was used. Mann–Whitney U and independent tests were used to compare NSS, NDS, and VAS scores between two groups at each time. In addition to the statistical methods mentioned earlier, the Nonparametric Wilcoxon rank sum test and the Binomial test (1-tailed) were used to assess possible differences in NSS, NDS, and VAS scores separately for each severity group. Furthermore, the Friedman test was used to compare NSS and VAS scores within each group over time. The STATA10 software was used for data analysis in this study (p < 0.05).

## Results

### Participant flow

**Fig. 2:**
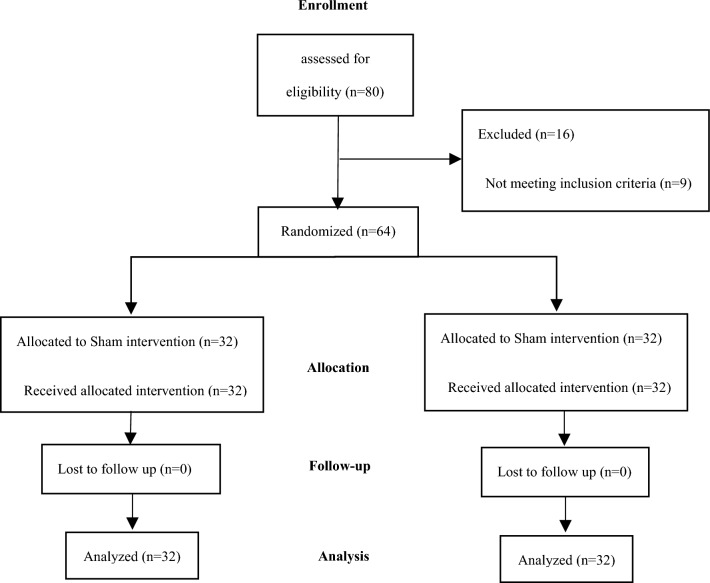
Participant flow diagram

A total of 80 participants were screened for the trial, of which nine participants did not meet the inclusion criteria, and seven participants declined to participate. Therefore, 64 participants were enrolled in the double-blind trial, with 32 participants assigned to each group (magnetic device group and sham device group). All participants completed the 12 week trial protocol and were included in the analyses, as shown in Fig. 2. Overall, the high completion rate of the trial (100%) suggests that the study protocol was well-accepted by the participants and that the magnetic and sham devices were well-tolerated. The large sample size (n = 64) further increases the statistical power of the study and helps to ensure that the results are reliable and valid.

### Baseline data

The obtained results indicate that both groups share similar characteristics, such as age, gender, and medical history (Table 2). The mean age of the patients was 59.83 ± 7.84 years old. In addition, 68.7% of the patients were women. Furthermore, it was founded that 53.12% of patients had taken medicine for DPN.

**Table 2:** Comparison of the demographic and medical characteristics between both groups

Variables	Magnet Group	Sham Group	*P* value^b^
	Mean ± SD^a^	Mean ± SD	
Age (y)	58.31 ± 9.25	61.34 ± 5.87	0.28
Weight (kg)	78.75 ± 13.45	75.40 ± 15.12	0.35
Height (cm)	163.53 ± 9.76	163.90 ± 8.44	0.93
BMI^c^ (kg/ m2)	29.52 ± 4.80	27.98 ± 4.58	0.19
Sex, No. (%)			
Males	10 (31.3)	10 (31.3)	1.00
Females	22 (68.7)	22 (68.7)	
Duration of incidence of diabetes (m)	165.03 ± 104.16	175.88 ± 87.73	0.53
Duration of the beginning of diabetic neuropathy (m)	63.88 ± 47.65	57.31 ± 43.02	0.68
Duration of exercise a week (h)	3.37 ± 3.43	2.92 ± 2.91	0.67
Pre-FBG^d^ (mg/dL)	170.88 ± 57.52	162.25 ± 56.08	0.54
Hb A1C (%)	8.04 ± 1.17	7.90 ± 1.10	0.61
Drugs for diabetes, No (%)			
Oral	16 (50)	17 (53.1)	0.96
Insulin	1 (3.1)	1 (3.1)	
Both	15 (46.9)	14 (43.8)	
Concomitant medications for neuropathy, No (%)			
None	14 (43.8)	16 (50)	0.84
Gabapentin 300 daily	3 (9.4)	3 (9.4)	
Vit B1 300 daily	3 (9.4)	2 (6.2)	
Vit D3 50000 monthly	6 (18.8)	6 (18.8)	
Vit B1 and Vit D3	3 (9.4)	4 (12.5)	
Gabapentin and Vit B1	2 (6.2)	0 (0)	
Gabapentin and Vit D3	0 (0)	1 (3.1)	
Gabapentin and Vit B1 and Vit D3	1 (3.1)	0 (0)	
Medical history, No (%)			
Blood pressure	15 (46.9)	17 (53.1)	0.62
Kidney diseases	5 (15.6)	3 (9.4)	0.70
Cardiac diseases	10 (31.2)	11 (34.4)	0.79
Mental disorders	2 (6.2)	1 (3.1)	1.00
Ocular diseases	17 (53.1)	14 (43.8)	0.45

^a^*SD* standard deviation, ^b^*P-value* probability value, ^c^*BMI* body mass index, ^d^*FBG* fasting blood glucose

### Outcomes

Table 3 shows the mean and standard deviations for primary and secondary outcomes in both groups at baseline and after treatment (4, 8, and 12 weeks).

**Table 3:** Comparison of outcome measure mean values in two groups

Outcome measure	Magnet (n = 32) Mean ± SD	Sham (n = 32) Mean ± SD	P value
NSS:			
Baseline	13 ± 1.88	12.63 ± 2.09	0.50
4 weeks	10.22 ± 2.82	12.66 ± 2.05	< 0.001^a^
8 weeks	6.25 ± 2.68	12.66 ± 2.05	< 0.001^a^
12 weeks	2.78 ± 2.61	12.50 ± 2.00	< 0.001^a^
VAS:			
Baseline	6.28 ± 2.30	5.47 ± 2.47	0.17
4 weeks	4.03 ± 2.20	5.34 ± 2.59	0.03^a^
8 weeks	2.50 ± 1.88	5.34 ± 2.54	< 0.001^a^
12 weeks	0.63 ± 0.94	5.28 ± 2.58	< 0.001^a^
NDS:			
Baseline	6.84 ± 1.83	6.53 ± 1.54	0.74
12 weeks	4.47 ± 1.36	6.53 ± 1.54	< 0.001^a^
NeuroQol:			
Baseline	66.97 ± 15.92	67.88 ± 16.65	0.17
12 weeks	35.56 ± 4.47	71.47 ± 15.46	< 0.001^a^

^a^Significance P value < 0.05

### NSS

The results of the study showed that there was a significant decrease (P < 0.001) in the mean scores for the magnetic device group, while there was no significant difference (P = 0.38) for the sham device group from baseline to week 12. All of the participants in both groups had severe neuropathy symptoms at baseline. Tables 4 and 5 present the number of participants with neuropathy symptoms as assessed by the NSS.

**Table 4:** Participants with neuropathy symptoms frequency in both groups at the baseline and 4, 8, and 12 weeks

Outcome measure		Baseline	4 weeks	8 weeks	12 weeks
Magnet (n = 32) No. (%)	Burning	30 (93.8)	25 (78.1)	17 (53.1)	10 (31.3)
	Numbness	27 (84.4)	19 (59.4)	7 (21.9)	2 (6.3)
	Paresthesia	30 (93.8)	23 (71.9)	10 (31.3)	4 (12.5)
	Feeling of weakness (fatigue, exhaustion)	24 (75)	11 (34.4)	4 (12.5)	0 (0)
	Cramps	27 (84.4)	12 (37.5)	4 (12.5)	0 (0)
	Pain	32 (100)	29 (90.6)	27 (84.4)	12 (37.5)
	Awakened from sleep	18 (56.3)	6 (18.8)	2 (6.3)	0 (0)
Sham (n = 32) No. (%)	Burning	31 (96.9)	31 (96.9)	31 (96.9)	31 (96.9)
	Numbness	27 (84.4)	27 (84.4)	26 (81.3)	26 (81.3)
	Paresthesia	30 (93.8)	30 (93.8)	30 (93.8)	30 (93.8)
	Feeling of weakness (fatigue, exhaustion)	26 (81.3)	25 (78.1)	24 (75)	24 (75)
	Cramps	19 (59.4)	19 (59.4)	19 (59.4)	19 (59.4)
	Pain	32 (100)	32 (100)	32 (100)	32 (100)
	Awakened from sleep	23 (71.9)	23 (71.9)	21 (65.6)	17 (53.1)

P value					
Burning			0.053	< 0.001^a^	< 0.001^a^
Numbness			0.050	< 0.001^a^	< 0.001^a^
Paresthesia			0.04^a^	< 0.001^a^	< 0.001^a^
Feeling of weakness (fatigue, exhaustion)			0.001^a^	< 0.001^a^	< 0.001^a^
Cramps			0.13	< 0.001^a^	< 0.001^a^
Pain			0.23	0.053	< 0.001^a^
Awakened from sleep			< 0.001^a^	< 0.001^a^	< 0.001^a^

^a^Significance P value < 0.05

**Table 5:** Participants with neuropathy symptoms frequency in both groups at the baseline and 4, 8, and 12 weeks

Outcome measure			Baseline	4 weeks	8 weeks	12 weeks
Magnet (n = 32) No. (%)	Localization	Feet	31 (96.9)	30 (93.8)	23 (71.9)	8 (25)
		Lower leg	1 (3.1)	2 (6.3)	7 (21.9)	11 (34.4)
		Elsewhere	0 (0)	0 (0)	2 (6.3)	13 (40.6)
	Exacerbation	Night	22 (68.8)	20 (62.5)	4 (12.5)	0 (0)
		Day	0 (0)	0 (0)	4 (12.5)	17 (53.1)
		Day and night	10 (31.3)	12 (37.5)	24 (75)	15 (46.9)
	Symptom improvement	Walking	11 (34.4)	10 (31.3)	5 (15.6)	2 (6.3)
		Standing	0 (0)	0 (0)	0 (0)	1 (3.1)
		Sitting or lying down	21 (65.6)	22 (68.8)	27 (84.4)	29 (90.6)
Sham (n = 32) No. (%)	Localization	Feet	31 (96.9)	31 (96.9)	31 (96.9)	31 (96.9)
		Lower leg	1 (3.1)	1 (3.1)	1 (3.1)	1 (3.1)
	Exacerbation	Night	14 (43.8)	15 (46.9)	20 (62.5)	19 (59.4)
		Day	1 (3.1)	0 (0)	0 (0)	0 (0)
		Day and night	17 (53.1)	17 (53.1)	12 (37.5)	13 (40.6)
	Symptom improvement	Walking	10 (31.3)	10 (31.3)	10 (31.3)	10 (31.3)
		Sitting or lying down	22 (68.8)	22 (68.8)	22 (68.8)	22 (68.8)

P value						
Localization				1.00	0.01^a^	< 0.001^a^
Exacerbation				0.31	< 0.001^a^	< 0.001^a^
Symptom improvement				1.00	0.23	0.02^a^

^a^Significance P value < 0.05

### VAS

At baseline, the mean VAS scores for the magnetic and sham device groups were 62/100 and 54/100 mm, respectively. The magnetic device group showed a greater reduction in VAS scores from baseline to week 12 compared to the sham device group, as shown in Table 3. Of the 64 patients who participated in the study, 12 (3 in the magnetic device group) had mild pain, 27 (14 in the magnetic device group) had moderate pain, 19 (11 in the magnetic device group) had severe pain, and 6 (4 in the magnetic device group) had the worst possible pain. These pain severity groups were determined based on the VAS scores reported by the participants. The results suggest that the magnetic device was effective in reducing pain severity in participants with a range of pain severity levels, including those with severe and worst possible pain.

### NDS

The results of the study showed that there was a significant decrease in mean scores for the magnetic device group, while there was no significant difference for the sham device group from baseline to week 12, as shown in Table 3. Of the 64 patients who participated in the study, 14 (7 in the magnetic device group) had mild neuropathy deficits, 44 (20 in the magnetic device group) had moderate neuropathy deficits, and 6 (5 in the magnetic device group) had severe neuropathy deficits. These neuropathy deficits were assessed using the NDS. The results suggest that the magnetic device was effective in reducing neuropathy deficits in participants with a range of severity levels, including those with moderate and severe neuropathy deficits. However, the lack of significant difference in the sham device group suggests that any observed improvements in the magnetic device group are unlikely to be due to placebo effects or natural recovery.

### QoL

At baseline, there was no significant difference between the magnetic and sham device groups. However, significant decreases in the mean scores for both groups were observed from baseline to week 12, as shown in Table 3. However, in the sham group, unlike the magnet group, this difference means patients had higher scores in Neuro-QoL and lower quality of life.

### Comparison of two groups during weeks

The results of the study showed that there were significant differences in the mean values of NSS and VAS in the magnetic device group, while no significant differences were observed in the sham device group, as shown in Table 6.

**Table 6:** Statistical significance values of NSS and VAS during weeks in both groups

Outcome measure	Magnet (n = 32) P value	Sham (n = 32) P value
NSS	< 0.001^a^	0.50
VAS	< 0.001^a^	0.14

^a^Significance P value < 0.05

### Harms

During the study, one patient (a woman) in the magnetic device group reported experiencing increased foot pain when wearing an ankle bracelet for 2 days. However, the pain decreased over time and the patient was able to continue using the device without further issues. In the sham device group, two patients (both men) reported experiencing erythema around the site due to an allergy to leather. No dropouts were registered due to adverse events. Overall, the low incidence of adverse events in the study suggests that the magnetic and sham devices were well-tolerated by the participants. The adverse events reported were generally mild and did not result in any serious complications or dropouts from the study.

## Discussion

The study results suggest that SMF therapy, which involves the use of a magnetic ankle bracelet, may be an effective treatment option for improving symptoms and quality of life in patients with type 2 diabetes over a period of 4 to 12 weeks. There is a growing body of research that has documented the therapeutic effect of SMF therapy in both experimental conditions and humans. To our knowledge, only one other study has investigated the effects of SMF therapy on DPN with neuropathic pain. In a previous double-blind, placebo-controlled study conducted by Weintraub and colleagues, a statistically significant therapeutic effect of SMF therapy on DPN with neuropathic pain was also observed [26]. However, there are several notable differences between Weintraub's study and the current study. Firstly, the current study found a significant therapeutic effect of SMF therapy within the first month of treatment, whereas Weintraub's study reported therapeutic effects only during the third and fourth months. This difference may be due to the use of different magnetic devices, as Weintraub’s participants used multipolar magnetic shoe insoles with a magnetic field strength of 45 mT, while the current study used a single bipolar magnetic ankle bracelet with a higher magnetic field strength. Secondly, the current study found a beneficial effect of SMF therapy on a range of DPN symptoms, whereas Weintraub's study was effective only on numbness, tingling, burning, and pain. This suggests that the magnetic ankle bracelet used in the current study may be a more versatile treatment option for individuals with DPN and neuropathic pain than the multipolar magnetic shoe insoles used in Weintraub's study. Overall, the results of both studies provide promising evidence for the potential therapeutic benefits of SMF therapy for DPN with neuropathic pain. However, further research with larger sample sizes and longer follow-up periods is needed to confirm these findings and to better understand the underlying mechanisms of action of SMF therapy on DPN symptoms and pain. Our findings are consistent with previous studies that reported the beneficial effects of SMF therapy on various conditions. For example, studies by Segal and Wolsko, and colleagues have shown that SMF therapy can lead to significant improvements in arthritis symptoms, including pain, stiffness, and range of motion [35, 36]. Segal and colleagues conducted a study using a quadrupolar static magnetic device with four permanent magnets delivering 190mT over each pole, and similar to our findings, they observed statistically significant therapeutic effects. However, in contrast to the Segal study, our study found that a therapeutic effect could be achieved with fewer magnetic fields and a smaller number of magnets, in the form of a single bipolar magnetic ankle bracelet with a magnetic field strength of 155 mT. This suggests that magnetic devices with fewer magnetic fields and magnets may be a more practical and convenient treatment option for patients, as well as potentially more cost-effective. However, it is important to note that more research is needed to determine the optimal parameters for SMF therapy, including the magnetic field strength, number of magnets, and duration of treatment, to maximize its therapeutic effects for various conditions. Alfano and colleagues conducted a study on the use of magnetic sleep pads delivering 395 mT in fibromyalgia patients [37]. They found a significant reduction in pain intensity during the third to sixth months of treatment. In contrast, our study found a positive effect of SMF therapy in the first month of treatment for DPN with neuropathic pain and used a lower magnetic field strength in the form of a single bipolar magnetic ankle bracelet with a magnetic field strength of 155 mT. These differences suggest that SMF therapy may have varying effects depending on the condition being treated, as well as the specific parameters of the magnetic device used. It is also worth noting that our study focused on DPN with neuropathic pain, while the Alfano study focused on fibromyalgia, a different condition with different symptoms and underlying mechanisms. Some authors reported the beneficial effect of SMF on wound healing [38, 39]. Despite the promising results of some studies, the efficacy of SMF therapy has been inconsistent across different conditions. For example, a double-blind, placebo-controlled, crossover pilot study conducted by Collacott and colleagues on 20 patients with low back pain found no statistically significant effect of SMF therapy [40]. The study used bipolar permanent magnets with a 30 mT flux density, applied for 18 h per day over the course of 1 week. These inconsistent findings may be due to differences in the study design, magnetic device parameters, or patient characteristics. It is also possible that the therapeutic effects of SMF therapy may vary depending on the specific condition being treated. Therefore, more research is needed to determine the optimal parameters and conditions for SMF therapy, as well as to identify which patient populations may benefit the most from this treatment modality. The results of a systematic review conducted by Pittler and colleagues suggested that the evidence for the efficacy of SMF therapy is not conclusive [24]. While some of the studies included in the review reported positive effects of SMF therapy, the overall quality of the evidence was deemed to be low or very low due to methodological limitations such as small sample sizes, inadequate blinding, and lack of standardized outcome measures. Therefore, the authors concluded that there is currently insufficient evidence to support the routine use of SMF therapy for the treatment of various conditions. Colbert and colleagues have argued that the conclusion of Pittler’s systematic review may have been unwarranted due to inadequate or inappropriate SMF dosing parameters in some of the studies included in the review [41]. They have proposed 10 essential dosing parameters for SMF therapy, including the physical design of the magnet, the distance of the magnet from the target tissue, magnet field strength, and dose, as well as study design factors such as the blinding and randomization of participants. Colbert and colleagues suggest that failure to properly control these dosing parameters may have contributed to the inconsistent results observed in previous studies of SMF therapy. For example, studies that have failed to demonstrate a beneficial effect of SMF therapy often used weaker magnetic fields (between 19 and 50 mT), which may not have been sufficient to produce a therapeutic effect. Therefore, it is important for future studies of SMF therapy to carefully consider and control these dosing parameters to maximize the potential therapeutic effects of this treatment modality. Additionally, more research is needed to determine the optimal dosing parameters for SMF therapy for various conditions, as well as to identify which patient populations may benefit the most from this treatment. Studies that have demonstrated a beneficial effect of SMF therapy typically used stronger magnetic fields, ranging from 47 to 180 mT. Consistent with these findings, our study also used a relatively strong magnetic field strength in the form of a single bipolar magnetic ankle bracelet with a magnetic field strength of 155 mT and found a positive effect of SMF therapy on DPN. These findings suggest that magnetic field strength is a significant factor in the therapeutic effects of SMF therapy. However, it is worth noting that the optimal magnetic field strength may vary depending on the specific condition being treated, as well as other factors such as the physical design of the magnet and the distance of the magnet from the target tissue. The therapeutic mechanisms of SMF therapy are not yet fully understood, but some studies have suggested that it may affect ion channel conduction properties and capsaicin-sensitive sensory fibers. Specifically, SMF may modulate the activity of voltage-gated ion channels, which play a key role in the transmission of pain signals and may also activate capsaicin-sensitive sensory fibers, which are involved in the perception of heat and pain [42, 43]. A study conducted by Okano and colleagues suggested that SMF therapy may affect ion channels related to C fibers, which may play a role in the transmission of pain signals. However, the precise mechanism by which SMF modulates these ion channels is not yet fully understood [44]. However, these proposed mechanisms are still speculative, and more research is needed to determine the underlying biological processes that mediate the therapeutic effects of SMF therapy. Additionally, the therapeutic effects of SMF therapy may likely involve multiple mechanisms, which may vary depending on the specific condition being treated and other factors such as the magnetic field strength and duration of treatment. Therefore, further research is needed to elucidate the precise mechanisms of action of SMF therapy to maximize its therapeutic effects for different conditions. To determine whether SMF therapy can produce permanent changes in biological processes, future studies may need to incorporate more sensitive biological markers. For example, microneurography and epidermal nerve fiber biopsy have been suggested as potential markers that could be used to assess the effects of SMF therapy on peripheral nerve function. These markers may provide more detailed information about the underlying biological processes that mediate the therapeutic effects of SMF therapy and may help to elucidate the potential mechanisms by which it produces its effects. Additionally, the use of these markers may help to determine whether SMF therapy can produce long-lasting changes in nerve function, which would be a key step in establishing its efficacy as a treatment modality [26]. Moreover, subgroup analysis identified that most of the sham group reported lower quality of life after 12 weeks. The greatest reduction in the pain level was reported in the magnet group in the 4th week after the intervention. Cramps and feelings of weakness responded more favorably than other symptoms to SMF therapy so after 12 weeks, all patients in the magnet group recovered. These findings suggest that SMF therapy may be a promising treatment modality, although further research is needed to confirm these effects and to determine the optimal parameters for this treatment. Therefore, future studies should consider incorporating longer follow-up periods and more detailed assessments of the quality of life and other symptoms to more fully understand the potential benefits and limitations of SMF therapy for various conditions.

Strengths of our study include randomized double-blind, placebo-controlled design, measurement of disability, use of validated scales, and the cooperative involvement of a diabetologist and physicist. Additionally, the study included monitoring of blood sugar levels,

Limitations of our study include the physical impossibility of blind ankle bracelets and we relied on patients’ self-report for an outcome.

In conclusion, SMF therapy can significantly reduce neuropathic symptoms and improve the quality of life in patients with type 2 diabetes. It was also found that using SMF therapy in medical treatment can help reduce the adverse effects of drugs. SMF therapy is a standard and widely accepted method with no reported complications by patients so far. Interestingly, it can also reduce the demand for specialist referrals, which can aid in making convenient healthcare policy decisions. Considering its safety and low cost, SMF therapy can help avoid the frequent prescription of expensive analgesic medications.

## Data Availability

The data used to support the findings of this study are included in the article. Further enquiries can be directed to the corresponding author.

## Abbreviations

DPN: Diabetic peripheral neuropathy
DM: Diabetes mellitus
TCAs: Tricyclic antidepressants
SMF: Static magnetic field
RCT: Randomized controlled trials
PEMFs: Pulsed electromagnetic fields
Qol: Quality of life
NSS: Neuropathy symptom score
VAS: Visual analogue scale
NDS: Neuropathic disability score
